# Dataset on reflection and transmission coefficients of ultrasonic shear horizontal guided waves in plates with wall thinning

**DOI:** 10.1016/j.dib.2018.11.053

**Published:** 2018-11-15

**Authors:** Alan C. Kubrusly, Miguel A. Freitas, Jean Pierre von der Weid, Steve Dixon

**Affiliations:** a Centre for Telecommunication Studies, Pontifical Catholic University of Rio de Janeiro, Rio de Janeiro, Brazil; b Department of Physics, University of Warwick, Coventry, U.K

## Abstract

This data article reports the data for reflection and transmission coefficients of the SH0 and SH1 ultrasonic guided waves modes due to their interaction with tapered wall thinning in aluminium plates. Several thinning depths and edge taper angles were machined, at the total of 35 different samples. Periodic permanent magnet array electromagnet acoustic transducers were used to generate and receive the waves. Both modes were individually generated and separated in the received signal by means of effective post-processing technique. Reflection and transmission coefficients were calculated at both the leading and trailing edges of the thinning region for mode-converted and non-mode converted signals; therefore, eight coefficients were calculated for each generated mode, at the total of sixteen coefficients for each sample. Additional finite-element model was used in order to obtain numerical values for the coefficients. These data were used in order to analyze the interaction of the SH0 and SH1 modes with wall thinning and the capabilities of using them in non-destructive evaluation of corrosion-like defects in the research paper entitled “Interaction of SH guided waves with wall thinning” (Kubrusly et al., 2019).

**Specifications table**

**Table:** 

Subject area	*Physics*
More specific subject area	*Non-destructive testing, ultrasonic wave propagation*
Type of data	*Tables, Figures*
How data were acquired	*Ultrasonic SH guided wave signals on machined aluminium plates. Ultrasound equipment: RITEC ® RPR-4000 Pulser/Receiver with ultrasound transducers: 10 mm pitch PPM EMATs from Sonemat Ltd.*
	*Additional finite element simulations performed with PZFlex*^*©*^*solver.*
Data format	*Filtered.*
Experimental factors	*Aluminium plates, 8 mm thick, 800 mm long, 250 mm wide, with machined thinning sections, 150 mm long all width wide, several depths and taper angles.*
Experimental features	*Ultrasound transducer positioned at the sample surface in specific positions in order to measure reflected and transmitted waves due to interaction with thinning region in the samples.*
Data source location	*Ultrasonics Group, Department of Physics, University of Warwick, Coventry CV4 7AL, United Kingdom.*
Data accessibility	*Data are with this article*
Related research article	*A. C. Kubrusly, M. A. Freitas, J. P. von der Weid, and S. Dixon, "Interaction of SH guided waves with wall thinning," NDT &E International, 101 (2019), pp. 94–103*[1]

**Value of the data**

• The data allow investigation on the interaction of the SH0 and SH1 guided wave modes with thinning regions that simulate wall loss due to corrosion in metallic plates, which is important for non-destructive tests of plates and pipes.
• Up to now, detailed experimental data on the reflection and transmission coefficients for mode-converted and non-mode converted waves of the SH0 and SH1 guided wave modes were not reported.
• The data allow one to address the capabilities and limitation on the use of ultrasonic SH guided wave to estimate and detect wall thinning.
• The data can be used for developing and evaluating novel techniques in order to assess the amount and severity of wall loss by means of reflected and transmitted SH guided waves.

## 1 Data

The dataset within this data article provides the reflection and transmission coefficients of shear horizontal (SH) guided wave modes at both the leading and trailing edges of linearly tapered thinning regions. Each experimental sample had a different thinning depth and taper angle, 35 different samples were machined and experimentally analyzed. Coefficients for reflection at the leading edge, transmission to the thinning region, reflection at the trailing edge, and transmission out of the thinning region, were calculated. Either the SH0 or SH1 modes were individually generated; both modes were received in each generation case for each coefficient in order to obtain data on mode-converted and non-mode converted waves, therefore giving rise to a total of 16 different coefficients for each sample. Additional numerical data were obtained by means of a finite-element model for a wider collection of thinning geometry. The coefficient data are reported in Table 1, Table 2, Table 3, Table 4, Table 5, Table 6, Table 7, Table 8 and in Table 9, Table 10, Table 11, Table 12, Table 13, Table 14, Table 15, Table 16, for generation of the SH0 and SH1 mode, respectively.

**Table 1:** *R*_00_ coefficient.

			Taper angle (degree)							
			90	65	55	45	35	30	25	10
Relative depth (%)	0	FEM	0.0054	0.0054	0.0054	0.0054	0.0054	0.0054	0.0054	0.0054
		Exp.	0.0342	0.0342	0.0342	0.0342	0.0342	0.0342	0.0342	0.0342
	12.5	FEM	0.0938	0.0850	0.0794	0.0783	0.0728	0.0648	0.0619	0.0122
		Exp.	0.0832	—	0.0783	0.0818	—	—	—	0.0280
	25	FEM	0.2266	0.1895	0.1735	0.1511	0.1122	0.0811	0.0417	0.0243
		Exp.	0.2054	—	0.1728	0.1373	—	—	—	0.0269
	37	FEM	0.3789	0.3287	0.2941	0.2469	0.1922	0.1736	0.1684	0.0165
		Exp.	0.3259	—	0.2805	0.2265	—	—	—	0.0354
	50	FEM	0.5104	0.4609	0.3980	0.3152	0.1941	0.1478	0.1203	0.0379
		Exp.	0.4660	—	0.3463	0.3001	—	—	—	0.0585
	62.5	FEM	0.6210	0.5355	0.4058	0.2558	0.1378	0.1439	0.1587	0.0447
		Exp.	0.5567	—	0.3211	0.2410	—	—	—	0.0648
	75	FEM	0.7243	0.5615	0.3373	0.1952	0.2358	0.1904	0.0745	0.0320
		Exp.	0.6702	—	0.2470	0.2003	0.1836	0.1628	0.0888	0.0794
	87.5	FEM	0.8450	0.4515	0.2628	0.3955	0.2381	0.0542	0.2358	0.0557
		Exp.	0.7524	0.4022	0.3136	0.2893	—	0.0947	0.1937	0.0843

**Table 2:** *R*_01_ coefficient.

			Taper angle (degree)							
			90	65	55	45	35	30	25	10
Relative depth (%)	0	FEM	0.0000	0.0000	0.0000	0.0000	0.0000	0.0000	0.0000	0.0000
		Exp.	0.0223	0.0223	0.0223	0.0223	0.0223	0.0223	0.0223	0.0223
	12.5	FEM	0.1311	0.1194	0.1126	0.1122	0.1041	0.0950	0.0925	0.0083
		Exp.	0.1019	—	0.0936	0.0983	—	—	—	0.0215
	25	FEM	0.2993	0.2551	0.2379	0.2136	0.1715	0.1357	0.0860	0.0324
		Exp.	0.2552	—	0.2165	0.1721	—	—	—	0.0294
	37	FEM	0.4595	0.4187	0.3920	0.3559	0.2947	0.2669	0.2680	0.0883
		Exp.	0.3747	—	0.3563	0.2803	—	—	—	0.0773
	50	FEM	0.4999	0.4719	0.4589	0.4567	0.4124	0.3672	0.3510	0.1246
		Exp.	0.4331	—	0.4207	0.3699	—	—	—	0.1143
	62.5	FEM	0.4693	0.4712	0.4961	0.4896	0.3885	0.3164	0.2836	0.1211
		Exp.	0.3902	—	0.4495	0.4082	—	—	—	0.1213
	75	FEM	0.3917	0.5067	0.5660	0.4989	0.3234	0.2876	0.3088	0.1255
		Exp.	0.3404	—	0.4928	0.4143	0.2751	0.2303	0.2374	0.1211
	87.5	FEM	0.2553	0.6435	0.6216	0.3804	0.3710	0.3536	0.2613	0.1320
		Exp.	0.2408	0.5904	0.4654	0.3494	—	0.3117	0.2037	0.1219

**Table 3:** *T*_00_ coefficient.

			Taper angle (degree)							
			90	65	55	45	35	30	25	10
Relative depth (%)	0	FEM	0.9997	0.9997	0.9997	0.9997	0.9997	0.9997	0.9997	0.9997
		Exp.	0.9918	0.9918	0.9918	0.9918	0.9918	0.9918	0.9918	0.9918
	12.5	FEM	0.9817	0.9839	0.9855	0.9854	0.9868	0.9881	0.9893	0.9945
		Exp.	0.9627	—	0.9851	0.9807	—	—	—	0.9871
	25	FEM	0.9099	0.9326	0.9395	0.9472	0.9579	0.9638	0.9695	0.9796
		Exp.	0.9092	—	0.9088	0.9205	—	—	—	0.9670
	37	FEM	0.7957	0.8328	0.8521	0.8727	0.8954	0.9051	0.9124	0.9867
		Exp.	0.7977	—	0.8269	0.8709	—	—	—	0.9667
	50	FEM	0.6992	0.7467	0.7869	0.8326	0.8915	0.9162	0.9331	0.9918
		Exp.	0.7047	—	0.7692	0.8105	—	—	—	0.9574
	62.5	FEM	0.6276	0.6969	0.7607	0.8304	0.9109	0.9384	0.9505	0.9912
		Exp.	0.6306	—	0.7437	0.7999	—	—	—	0.9575
	75	FEM	0.5677	0.6501	0.7438	0.8395	0.9193	0.9374	0.9533	0.9917
		Exp.	0.5420	—	0.7155	0.7699	0.8649	0.8210	0.8292	0.9459
	87.5	FEM	0.4733	0.6099	0.7277	0.8371	0.8964	0.9327	0.9404	0.9900
		Exp.	0.4066	0.5450	0.6579	0.7288	—	0.7640	0.7787	0.8235

**Table 4:** *T*_01_ coefficient.

			Taper angle (degree)							
			90	65	55	45	35	30	25	10
Relative depth (%)	0	FEM	0.0000	0.0000	0.0000	0.0000	0.0000	0.0000	0.0000	0.0000
		Exp.	0.0486	0.0486	0.0486	0.0486	0.0486	0.0486	0.0486	0.0486
	12.5	FEM	0.0657	0.0624	0.0596	0.0616	0.0601	0.0601	0.0596	0.0630
		Exp.	0.1186	—	0.0857	0.1097	—	—	—	0.0922
	25	FEM	0.0713	0.0694	0.0706	0.0734	0.0776	0.0816	0.0862	0.0748
		Exp.	0.0906	—	0.0855	0.0961	—	—	—	0.0812
	37	FEM	0.0333	0.0361	0.0444	0.0573	0.0643	0.0645	0.0611	0.0368
		Exp.	0.0424	—	0.0454	0.0377	—	—	—	0.0522
	50	FEM	0.0045	0.0040	0.0034	0.0027	0.0021	0.0020	0.0017	0.0007
		Exp.	0.0259	—	0.0207	0.0305	—	—	—	0.0445
	62.5	FEM	0.0000	0.0001	0.0001	0.0000	0.0000	0.0000	0.0000	0.0000
		Exp.	0.0333	—	0.0423	0.0471	—	—	—	0.0442
	75	FEM	0.0001	0.0000	0.0000	0.0000	0.0000	0.0000	0.0000	0.0000
		Exp.	0.0306	—	0.0552	0.0586	0.1027	0.0644	0.0697	0.0672
	87.5	FEM	0.0000	0.0000	0.0000	0.0000	0.0000	0.0000	0.0000	0.0000
		Exp.	0.0462	0.0437	0.1001	0.0813	—	0.0555	0.0715	0.1109

**Table 5:** *TR*_00_ coefficient.

			Taper angle (degree)							
			90	65	55	45	35	30	25	10
Relative depth (%)	0	FEM	0.0028	0.0028	0.0028	0.0028	0.0028	0.0028	0.0028	0.0028
		Exp.	0.0190	0.0190	0.0190	0.0190	0.0190	0.0190	0.0190	0.0190
	12.5	FEM	0.0622	0.0539	0.0533	0.0506	0.0471	0.0452	0.0399	0.0116
		Exp.	0.0579	—	0.0497	0.0533	—	—	—	0.0221
	25	FEM	0.0854	0.0755	0.0674	0.0539	0.0323	0.0237	0.0106	0.0188
		Exp.	0.0888	—	0.0514	0.0517	—	—	—	0.0234
	37	FEM	0.0451	0.0305	0.0201	0.0202	0.0275	0.0366	0.0419	0.0216
		Exp.	0.0483	—	0.0424	0.0367	—	—	—	0.0374
	50	FEM	0.0253	0.0207	0.0324	0.0481	0.0630	0.0558	0.0223	0.0264
		Exp.	0.0424	—	0.0549	0.0480	—	—	—	0.0439
	62.5	FEM	0.0462	0.0749	0.0917	0.0987	0.0541	0.0325	0.0709	0.0225
		Exp.	0.0616	—	0.0961	0.0928	—	—	—	0.0484
	75	FEM	0.1214	0.1495	0.1661	0.1263	0.0787	0.1301	0.0634	0.0279
		Exp.	0.1229	—	0.1410	0.0958	0.0684	0.0897	0.0694	0.0552
	87.5	FEM	0.2318	0.2557	0.2080	0.1839	0.2118	0.1591	0.1760	0.0709
		Exp.	0.1765	0.1834	0.1385	0.0865	—	0.0863	0.1327	0.0462

**Table 6:** *TR*_01_ coefficient.

			Taper angle (degree)							
			90	65	55	45	35	30	25	10
Relative depth (%)	0	FEM	0.0000	0.0000	0.0000	0.0000	0.0000	0.0000	0.0000	0.0000
		Exp.	0.0409	0.0409	0.0409	0.0409	0.0409	0.0409	0.0409	0.0409
	12.5	FEM	0.0688	0.0603	0.0590	0.0568	0.0539	0.0514	0.0468	0.0080
		Exp.	0.0688	—	0.0819	0.0655	—	—	—	0.0150
	25	FEM	0.0855	0.0775	0.0702	0.0584	0.0428	0.0335	0.0208	0.0141
		Exp.	0.0844	—	0.0549	0.0602	—	—	—	0.0346
	37	FEM	—	—	—	—	—	—	—	—
		Exp.	—	—	—	—	—	—	—	—
	50	FEM	—	—	—	—	—	—	—	—
		Exp.	—	—	—	—	—	—	—	—
	62.5	FEM	—	—	—	—	—	—	—	—
		Exp.	—	—	—	—	—	—	—	—
	75	FEM	—	—	—	—	—	—	—	—
		Exp.	—	—	—	—	—	—	—	—
	87.5	FEM	—	—	—	—	—	—	—	—
		Exp.	—	—	—	—	—	—	—	—

**Table 7:** *TT*_00_ coefficient.

			Taper angle (degree)							
			90	65	55	45	35	30	25	10
Relative depth (%)	0	FEM	0.9999	0.9999	0.9999	0.9999	0.9999	0.9999	0.9999	0.9999
		Exp.	1.0218	1.0218	1.0218	1.0218	1.0218	1.0218	1.0218	1.0218
	12.5	FEM	0.9636	0.9704	0.9714	0.9729	0.9750	0.9769	0.9790	0.9903
		Exp.	0.9840	—	1.0085	1.0100	—	—	—	1.0062
	25	FEM	0.8280	0.8646	0.8796	0.8980	0.9179	0.9282	0.9390	0.9575
		Exp.	0.8523	—	0.8832	0.9168	—	—	—	0.9768
	37	FEM	0.6327	0.6956	0.7289	0.7632	0.8021	0.8202	0.8326	0.9729
		Exp.	0.6546	—	0.7330	0.7978	—	—	—	0.9656
	50	FEM	0.4885	0.5638	0.6205	0.7017	0.7947	0.8397	0.8741	0.9822
		Exp.	0.5171	—	0.6507	0.7324	—	—	—	0.9780
	62.5	FEM	0.3942	0.4819	0.5748	0.6986	0.8297	0.8812	0.9055	0.9835
		Exp.	0.4097	—	0.6196	0.7013	—	—	—	0.9888
	75	FEM	0.3223	0.4259	0.5524	0.7120	0.8442	0.8771	0.9102	0.9824
		Exp.	0.3232	—	0.6193	0.7144	0.8446	0.8646	0.9094	0.9745
	87.5	FEM	0.2243	0.3699	0.5331	0.7049	0.8035	0.8688	0.8849	0.9778
		Exp.	0.2449	0.3837	0.6299	0.7267	—	0.9013	0.8958	0.9882

**Table 8:** *TT*_01_ coefficient.

			Taper angle (degree)							
			90	65	55	45	35	30	25	10
Relative depth (%)	0	FEM	0.0000	0.0000	0.0000	0.0000	0.0000	0.0000	0.0000	0.0000
		Exp.	0.0458	0.0458	0.0458	0.0458	0.0458	0.0458	0.0458	0.0458
	12.5	FEM	0.1307	0.1276	0.1305	0.1258	0.1253	0.1263	0.1221	0.1115
		Exp.	0.1000	—	0.1920	0.1432	—	—	—	0.0968
	25	FEM	0.3152	0.2912	0.2784	0.2598	0.2519	0.2473	0.2416	0.2248
		Exp.	0.3429	—	0.2599	0.2739	—	—	—	0.2321
	37	FEM	0.4622	0.4411	0.4278	0.4130	0.3921	0.3779	0.3717	0.1643
		Exp.	0.4469	—	0.4082	0.4167	—	—	—	0.2004
	50	FEM	0.4932	0.4840	0.4780	0.4430	0.3988	0.3632	0.3297	0.1288
		Exp.	0.5096	—	0.4329	0.4392	—	—	—	0.1642
	62.5	FEM	0.4796	0.4904	0.4826	0.4344	0.3723	0.3238	0.2819	0.1272
		Exp.	0.5014	—	0.4248	0.4310	—	—	—	0.1753
	75	FEM	0.4415	0.4592	0.4639	0.4242	0.3548	0.3036	0.2777	0.1273
		Exp.	0.4577	—	0.4193	0.4256	0.3387	0.3203	0.2619	0.1652
	87.5	FEM	0.3396	0.4054	0.4433	0.4136	0.3354	0.3013	0.2703	0.1269
		Exp.	0.3595	0.3975	0.4022	0.4061	—	0.2647	0.2749	0.1585

**Table 9:** *R*_11_ coefficient.

			Taper angle (degree)							
			90	65	55	45	35	30	25	10
Relative depth (%)	0	FEM	0.0093	0.0093	0.0093	0.0093	0.0093	0.0093	0.0093	0.0093
		Exp.	0.0187	0.0187	0.0187	0.0187	0.0187	0.0187	0.0187	0.0187
	12.5	FEM	0.2104	0.1791	0.1760	0.1675	0.1520	0.1384	0.1224	0.0192
		Exp.	0.1716	—	0.1572	0.1521	—	—	—	0.0263
	25	FEM	0.4484	0.3523	0.3071	0.2539	0.1769	0.1218	0.0628	0.0277
		Exp.	0.3964	—	0.2670	0.2288	—	—	—	0.0222
	37	FEM	0.5254	0.4055	0.3325	0.2295	0.0918	0.1164	0.1779	0.1222
		Exp.	0.4762	—	0.3054	0.2301	—	—	—	0.0762
	50	FEM	0.5261	0.5235	0.5004	0.5261	0.5827	0.6194	0.6949	0.9014
		Exp.	0.4927	—	0.5067	0.4084	—	—	—	0.6858
	62.5	FEM	0.4801	0.4968	0.5179	0.5095	0.6167	0.7079	0.8474	0.9786
		Exp.	0.4444	—	0.5072	0.4449	—	—	—	0.7662
	75	FEM	0.5512	0.4369	0.4686	0.5275	0.6927	0.7762	0.8562	0.9794
		Exp.	0.5341	—	0.4842	0.4778	0.6059	0.6462	0.7337	0.7579
	87.5	FEM	0.7193	0.2709	0.4839	0.6566	0.7045	0.7241	0.8770	0.9772
		Exp.	0.6338	0.2566	0.5652	0.5645	—	0.6432	0.7261	0.7516

**Table 10:** *R*_10_ coefficient.

			Taper angle (degree)							
			90	65	55	45	35	30	25	10
Relative depth (%)	0	FEM	0.0000	0.0000	0.0000	0.0000	0.0000	0.0000	0.0000	0.0000
		Exp.	0.0080	0.0080	0.0080	0.0080	0.0080	0.0080	0.0080	0.0080
	12.5	FEM	0.0681	0.0577	0.0568	0.0537	0.0483	0.0433	0.0375	0.0091
		Exp.	0.0392	—	0.0399	0.0381	—	—	—	0.0072
	25	FEM	0.1598	0.1235	0.1071	0.0858	0.0553	0.0329	0.0136	0.0160
		Exp.	0.0975	—	0.0679	0.0570	—	—	—	0.0132
	37	FEM	0.2087	0.1538	0.1221	0.0788	0.0410	0.0646	0.0796	0.0218
		Exp.	0.1326	—	0.0875	0.0593	—	—	—	0.0178
	50	FEM	0.2298	0.2023	0.1900	0.2007	0.2076	0.2130	0.1996	0.0435
		Exp.	0.1530	—	0.1342	0.1069	—	—	—	0.0717
	62.5	FEM	0.2146	0.2066	0.2076	0.2060	0.2216	0.2163	0.1757	0.0713
		Exp.	0.1328	—	0.1582	0.1323	—	—	—	0.0994
	75	FEM	0.1740	0.2246	0.2380	0.2200	0.1972	0.1845	0.1777	0.0702
		Exp.	0.1052	—	0.1813	0.1437	0.1469	0.1332	0.1399	0.0869
	87.5	FEM	0.1089	0.2812	0.2456	0.1693	0.1958	0.2284	0.1545	0.0767
		Exp.	0.0582	0.2069	0.1560	0.1182	—	0.1555	0.1164	0.0886

**Table 11:** *T*_11_ coefficient.

			Taper angle (degree)							
			90	65	55	45	35	30	25	10
Relative depth (%)	0	FEM	1.0123	1.0123	1.0123	1.0123	1.0123	1.0123	1.0123	1.0123
		Exp.	0.9617	0.9617	0.9617	0.9617	0.9617	0.9617	0.9617	0.9617
	12.5	FEM	0.9426	0.9588	0.9596	0.9625	0.9684	0.9722	0.9761	0.9894
		Exp.	0.9153	—	0.9410	0.9192	—	—	—	0.9463
	25	FEM	0.5191	0.6274	0.6614	0.6903	0.7195	0.7321	0.7379	0.7449
		Exp.	0.4953	—	0.5632	0.6268	—	—	—	0.6685
	37	FEM	0.0825	0.1447	0.1632	0.1781	0.1855	0.1858	0.1863	0.2092
		Exp.	0.1000	—	0.1734	0.1785	—	—	—	0.2182
	50	FEM	0.0060	0.0157	0.0183	0.0202	0.0215	0.0229	0.0264	0.0354
		Exp.	0.0149	—	0.0105	0.0233	—	—	—	0.0221
	62.5	FEM	0.0000	0.0000	0.0000	0.0000	0.0000	0.0000	0.0000	0.0000
		Exp.	0.0211	—	0.0145	0.0193	—	—	—	0.0059
	75	FEM	0.0000	0.0000	0.0000	0.0000	0.0000	0.0000	0.0000	0.0000
		Exp.	0.0206	—	0.0196	0.0217	0.0223	0.0130	0.0114	0.0077
	87.5	FEM	0.0000	0.0000	0.0000	0.0000	0.0000	0.0000	0.0000	0.0000
		Exp.	0.0272	0.0227	0.0259	0.0235	—	0.0098	0.0103	0.0106

**Table 12:** *T*_10_ coefficient.

			Taper angle (degree)							
			90	65	55	45	35	30	25	10
Relative depth (%)	0	FEM	0.0018	0.0018	0.0018	0.0018	0.0018	0.0018	0.0018	0.0018
		Exp.	0.0563	0.0563	0.0563	0.0563	0.0563	0.0563	0.0563	0.0563
	12.5	FEM	0.0727	0.0634	0.0628	0.0615	0.0579	0.0565	0.0549	0.0494
		Exp.	0.0989	—	0.0792	0.0910	—	—	—	0.0717
	25	FEM	0.1850	0.1516	0.1396	0.1306	0.1187	0.1151	0.1119	0.0979
		Exp.	0.1495	—	0.0995	0.1122	—	—	—	0.0790
	37	FEM	0.2673	0.2271	0.2135	0.2063	0.1963	0.1958	0.1909	0.1133
		Exp.	0.2267	—	0.1689	0.1811	—	—	—	0.1023
	50	FEM	0.3183	0.3027	0.2903	0.2863	0.2756	0.2635	0.2318	0.0510
		Exp.	0.2936	—	0.2186	0.2476	—	—	—	0.0811
	62.5	FEM	0.3426	0.3379	0.3179	0.3000	0.2627	0.2354	0.1853	0.0722
		Exp.	0.3033	—	0.2177	0.2438	—	—	—	0.0730
	75	FEM	0.3395	0.3450	0.3163	0.2873	0.2408	0.2182	0.1760	0.0715
		Exp.	0.2908	—	0.2021	0.2232	0.1709	0.1405	0.1122	0.0695
	87.5	FEM	0.3001	0.3371	0.3086	0.2716	0.2376	0.2147	0.1761	0.0717
		Exp.	0.2126	0.2126	0.1607	0.1839	—	0.1245	0.1023	0.0562

**Table 13:** *TR*_11_ coefficient.

			Taper angle (degree)							
			90	65	55	45	35	30	25	10
Relative depth (%)	0	FEM	0.0121	0.0121	0.0121	0.0121	0.0121	0.0121	0.0121	0.0121
		Exp.	0.0316	0.0316	0.0316	0.0316	0.0316	0.0316	0.0316	0.0316
	12.5	FEM	0.2230	0.2023	0.2023	0.1870	0.1713	0.1526	0.1443	0.0328
		Exp.	0.1874	—	0.1688	0.1692	—	—	—	0.0232
	25	FEM	0.2871	0.2702	0.2387	0.2103	0.1374	0.0885	0.0412	0.0403
		Exp.	0.2542	—	0.2007	0.1701	—	—	—	0.0409
	37	FEM	0.0522	0.0588	0.0562	0.0619	0.0744	0.0709	0.0591	0.0549
		Exp.	0.0532	—	0.0848	0.0566	—	—	—	0.0612
	50	FEM	—	—	—	—	—	—	—	—
		Exp.	—	—	—	—	—	—	—	—
	62.5	FEM	—	—	—	—	—	—	—	—
		Exp.	—	—	—	—	—	—	—	—
	75	FEM	—	—	—	—	—	—	—	—
		Exp.	—	—	—	—	—	—	—	—
	87.5	FEM	—	—	—	—	—	—	—	—
		Exp.	—	—	—	—	—	—	—	—

**Table 14:** *TR*_10_ coefficient.

			Taper angle (degree)							
			90	65	55	45	35	30	25	10
Relative depth (%)	0	FEM	0.0000	0.0000	0.0000	0.0000	0.0000	0.0000	0.0000	0.0000
		Exp.	0.0128	0.0128	0.0128	0.0128	0.0128	0.0128	0.0128	0.0128
	12.5	FEM	0.0589	0.0531	0.0527	0.0478	0.0439	0.0388	0.0361	0.0108
		Exp.	0.0539	—	0.0402	0.0462	—	—	—	0.0135
	25	FEM	0.0875	0.0776	0.0667	0.0561	0.0330	0.0176	0.0096	0.0149
		Exp.	0.0779	—	0.0576	0.0422	—	—	—	0.0124
	37	FEM	0.0395	0.0432	0.0317	0.0160	0.0250	0.0313	0.0302	0.0148
		Exp.	0.0318	—	0.0278	0.0185	—	—	—	0.0179
	50	FEM	0.0137	0.0158	0.0162	0.0179	0.0127	0.0092	0.0108	0.0090
		Exp.	0.0267	—	0.0245	0.0252	—	—	—	0.0153
	62.5	FEM	0.0413	0.0462	0.0398	0.0249	0.0125	0.0173	0.0114	0.0062
		Exp.	0.0323	—	0.0235	0.0203	—	—	—	0.0105
	75	FEM	0.1167	0.0841	0.0494	0.0240	0.0292	0.0196	0.0131	0.0072
		Exp.	0.0855	—	0.0236	0.0173	0.0240	0.0170	0.0107	0.0108
	87.5	FEM	0.1791	0.0849	0.0663	0.0648	0.0439	0.0350	0.0254	0.0102
		Exp.	0.1025	0.0609	0.0240	0.0353	—	0.0176	0.0216	0.0088

**Table 15:** *TT*_10_ coefficient.

			Taper angle (degree)							
			90	65	55	45	35	30	25	10
Relative depth (%)	0	FEM	0.0018	0.0018	0.0018	0.0018	0.0018	0.0018	0.0018	0.0018
		Exp.	0.0193	0.0193	0.0193	0.0193	0.0193	0.0193	0.0193	0.0193
	12.5	FEM	0.0921	0.0845	0.0846	0.0831	0.0802	0.0795	0.0795	0.0825
		Exp.	0.0770	—	0.0519	0.0755	—	—	—	0.0585
	25	FEM	0.1594	0.1375	0.1291	0.1220	0.1118	0.1154	0.1179	0.0996
		Exp.	0.1383	—	0.0989	0.1163	—	—	—	0.0810
	37	FEM	0.2070	0.1926	0.1860	0.1831	0.1755	0.1750	0.1714	0.1094
		Exp.	0.1755	—	0.1490	0.1649	—	—	—	0.0956
	50	FEM	0.2307	0.2286	0.2244	0.2202	0.2187	0.2161	0.2012	0.0510
		Exp.	0.2097	—	0.1749	0.2009	—	—	—	0.0827
	62.5	FEM	0.2232	0.2238	0.2192	0.2146	0.2116	0.2016	0.1707	0.0716
		Exp.	0.1942	—	0.1696	0.1893	—	—	—	0.0715
	75	FEM	0.1884	0.2010	0.2120	0.2102	0.2003	0.1919	0.1634	0.0710
		Exp.	0.1628	—	0.1702	0.1856	0.1625	0.1513	0.1290	0.0679
	87.5	FEM	0.1316	0.1944	0.2097	0.1942	0.1975	0.1866	0.1632	0.0710
		Exp.	0.1194	0.1495	0.1543	0.1609	—	0.1400	0.1218	0.0674

**Table 16:** *TT*_11_ coefficient.

			Taper angle (degree)							
			90	65	55	45	35	30	25	10
Relative depth (%)	0	FEM	1.0198	1.0198	1.0198	1.0198	1.0198	1.0198	1.0198	1.0198
		Exp.	0.9868	0.9868	0.9868	0.9868	0.9868	0.9868	0.9868	0.9868
	12.5	FEM	0.9207	0.9454	0.9443	0.9498	0.9617	0.9703	0.9752	0.9985
		Exp.	0.9227	—	0.9460	0.9361	—	—	—	0.9790
	25	FEM	0.5001	0.6831	0.7545	0.8051	0.8751	0.9021	0.9174	0.9411
		Exp.	0.5163	—	0.7294	0.7882	—	—	—	0.9366
	37	FEM	0.3338	0.3937	0.4888	0.5659	0.5983	0.5905	0.5834	0.7127
		Exp.	0.3072	—	0.4294	0.5354	—	—	—	0.6460
	50	FEM	0.4868	0.4430	0.4174	0.4137	0.3848	0.3412	0.2615	0.0106
		Exp.	0.4939	—	0.3760	0.3948	—	—	—	0.0234
	62.5	FEM	0.5626	0.5582	0.5103	0.4675	0.3623	0.2767	0.1657	0.0242
		Exp.	0.5675	—	0.4187	0.4410	—	—	—	0.0421
	75	FEM	0.5429	0.5831	0.5084	0.4296	0.3095	0.2387	0.1483	0.0238
		Exp.	0.5402	—	0.4087	0.4316	0.2897	0.2242	0.1379	0.0381
	87.5	FEM	0.4154	0.5717	0.4820	0.3788	0.3009	0.2302	0.1493	0.0238
		Exp.	0.4570	0.5375	0.3992	0.3886	—	0.1846	0.1309	0.0369

## 2 Experimental design, materials and methods

**Fig. 1:**
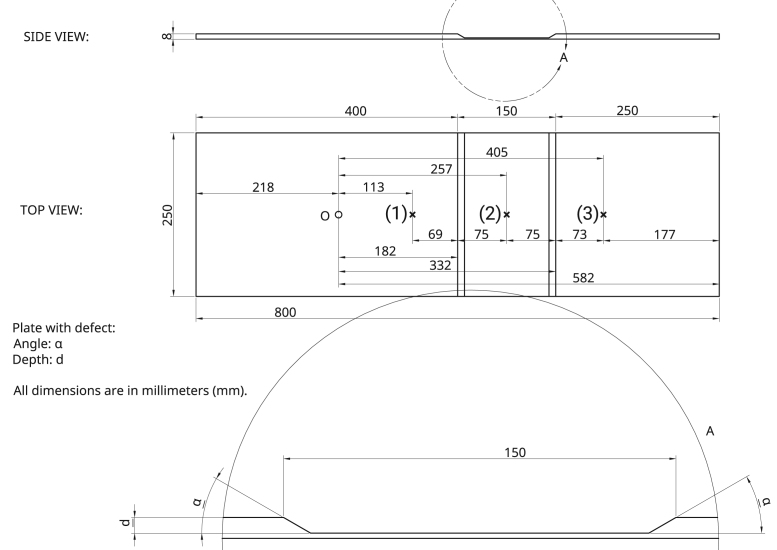
Plate dimension with a machined section. Marked positions denote the origin *O*, where transmitter is positioned, and receiver positions before the thinning region (1), in the middle of the region (2) and after the region (3).

**Fig. 2:**
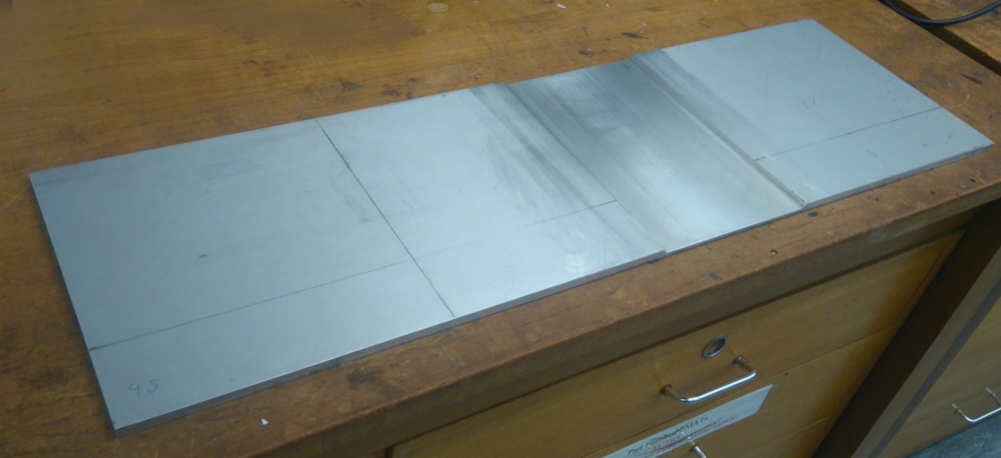
Machined plate at 45° edges and 4 mm depth.

Aluminium plates were used as test samples with dimension of 8 mm thick, 800 mm long and 250 mm wide. A tapered thinner section was machined in each sample starting at position $\ell_{a}$ = 182 mm with a total length of $\ell_{d}$ = 150 mm, several different depths, *d*, and edge angles, *α*, of the thinned region were machined in order to analyze the coefficients as a function of *d* and *α*. Specimens were prepared at edge angles of 10°, 45°, 55°, and 90°; for each of these angles, depths from 1 mm down to 7 mm were machined in 1 mm step. Additional specimens were prepared with 6 mm and 7 mm depth at edge angles of 25°, 30° and 35° and 25°, 30° and 65°, respectively. Therefore, a total of 34 samples were machined plus one non-machined reference sample, all of which were experimentally evaluated. Fig. 1 shows the sample and machined thinning region drawing with dimension and Fig. 2 shows one machined test sample.

The machined samples were experimentally evaluated using a RITEC ® RPR-4000 Pulser/Receiver and periodic permanent magnet array electromagnet acoustic transducers (PPM EMATs) from Sonemat Ltd. (3cycle, 10 mm nominal wavelength) as transmitter and receiver. PPM EMATs are able to generate shear horizontal guided waves in metallic plates [2]. In order to generate either the SH0 or the SH1 mode an 8 cycle tone burst at 311 kHz or 367 kHz, respectively, were applied to the transmitter PPM EMAT according to dispersion curve of each mode [1]. Dual excitation and reception on both plate׳s surfaces was adopted in order to ensure that a single mode was generated and then to separate the two possible received modes due to mode conversion. Details on the dual transduction procedure and experimental setup are described in Refs. [3] and [1], respectively.

Transmitters were placed at position *O* whereas receiver was positioned at positions (1), (2) or (3), see Fig. 1, in order to receive signals before, at and after the thinning region, respectively. Signals acquired by the oscilloscope were averaged in order to diminish the noise level, also a digital low-pass filter at 400 kHz 3 dB cut-off frequency was applied to the raw signals. Signals acquired at both surfaces were combined following [3] in order to separate mode-converted and non-mode converted signals for each generation.

Four coefficients were calculated, namely *R*_*ij*_*, T*_*ij*_*, TR*_*ij*_*, TT*_*ij*_, which denote the reflection at the thinning region leading edge, transmission to the region, reflection at the thinning region trailing edge, and transmission out of the far end of the region, respectively. The first subscript, *i*, denotes the generated mode, whereas the second one, *j*, denotes the received mode. Either *i* or *j* can be 0 or 1, here, corresponding to the SH0 or SH1 modes, respectively. These coefficients are defined by:

(1a) $$R_{\mathrm{ij}}=\frac{A_{j}^{\left(1\right)-}}{A_{i}^{\left(1\right)+}},$$

(1b) $$T_{\mathrm{ij}}=\frac{A_{j}^{\left(2\right)+}}{A_{i}^{\left(1\right)+}}\sqrt{\frac{h-d}{h}},$$

(1c) $${\mathrm{TR}}_{\mathrm{ij}}=\frac{A_{j}^{\left(2\right)-}}{A_{i}^{\left(1\right)+}}\sqrt{\frac{h-d}{h}},$$

(1d) $${\mathrm{TT}}_{\mathrm{ij}}=\frac{A_{j}^{\left(3\right)+}}{A_{i}^{\left(1\right)+}},$$

where A is the peak-to-peak of the received signal inside a time gate in which the mode is expected to arrive, the superscripts + and – mean the forward and backward propagating waves, and (1), (2) and (3) indicate the reading positions according to Fig. 1. All coefficients are related to the incident wave, $A_{i}^{\left(1\right)+}$. Since the wave amplitude is increased when it is transmitted to a thinner region, due to the energy distribution across the thickness, it is necessary to include the square root in Eq. (1b) and (1c) in order to compensate it, where h is the plate׳s original thickness, and therefore h−d is the remaining thickness in the thinner region.

The time gate to select the amplitude $A_{i}^{\left(1\right)+}$ of the incident mode i before the thinning region, starts and ends, respectively at:

(2a) $${t_{1}}_{i}^{\left(1\right)+}=\frac{x^{\left(1\right)}}{c_{g_{i}}}-\Delta_{i},$$

(2b) $${t_{2}}_{i}^{\left(1\right)+}=\frac{x^{\left(1\right)}}{c_{g_{i}}}+\frac{N}{f_{c}}+\Delta_{i},$$

where $x^{\left(1\right)}$ is the longitudinal coordinate of position (1), $c_{g_{i}}$ is the group velocity of the generated mode, i, at its working frequency, $f_{c}$, N is the number of cycles used in the exciting signal and $\Delta_{i}$ is a time margin which ensures that the whole signal is included in the time gate, empirically $\Delta_{i}=2^{i}N/{4f}_{c}$. The group velocities for the SH0 and SH1 modes in an 8 mm aluminium plate are $c_{g_{0}}=$ 3111 m/s and $c_{g_{1}}=$ 2428 m/s, respectively at 311 kHz and 367 kHz. The time gates to select the other amplitudes, namely, $A_{j}^{\left(1\right)-}$, $A_{j}^{\left(2\right)+}$, $A_{j}^{\left(2\right)-}$and $A_{j}^{\left(3\right)+}$ due to the incident mode i, start and end instants are, respectively:

(3a) $${t_{1}}_{\mathrm{ij}}^{\left(1\right)-}=\frac{\ell_{a}}{c_{g_{i}}}+\frac{{\ell_{a}-x}^{\left(1\right)}}{c_{g_{j}}}-\Delta_{i},$$

(3b) $${t_{2}}_{\mathrm{ij}}^{\left(1\right)-}\;=\;\frac{\ell_{a}+d\;\cot\;\left(\alpha \right)}{c_{g_{i}}}\;+\;\frac{\ell_{a}+d\;\cot\;\left(\alpha \right){-x}^{\left(1\right)}}{c_{g_{j}}}\;+\;\frac{N}{f_{c}}\;+\;\Delta_{i},$$

(4a) $${t_{1}}_{\mathrm{ij}}^{\left(2\right)+}\;=\;\frac{\ell_{a}}{c_{g_{i}}}\;+\;\frac{x^{\left(2\right)}-\ell_{a}}{c_{g_{j}}(h-d)}\;-\;\Delta_{i},$$

(4b) $${t_{2}}_{\mathrm{ij}}^{\left(2\right)+}\;=\;\frac{\ell_{a}}{c_{g_{i}}}\;+\;\frac{x^{\left(2\right)}-\ell_{a}}{c_{g_{j}}(h-d)}\;+\;\frac{N}{f_{c}}\;+\;\Delta_{i},$$

(5a) $${t_{1}}_{\mathrm{ij}}^{\left(2\right)-}\;=\;\frac{\ell_{a}}{c_{g_{i}}}\;+\;\frac{{2\ell }_{d}+{\ell_{a}-x}^{\left(2\right)}}{{\max\{c}_{g_{j}}(h-d),c_{g_{i}}(h-d)\}}-\Delta_{i},$$

(5b) $${t_{2}}_{\mathrm{ij}}^{\left(2\right)-}=\frac{\ell_{a}}{c_{g_{i}}}\;+\;\frac{{2\ell }_{d}+{\ell_{a}-x}^{\left(2\right)}}{{\min\{c}_{g_{j}}(h-d),c_{g_{i}}(h-d)\}}\;+\;\frac{N}{f_{c}}\;+\;\Delta_{i},$$

(6a) $${t_{1}}_{\mathrm{ij}}^{\left(3\right)+}=\frac{\ell_{a}}{c_{g_{i}}}\;+\;\frac{\ell_{d}}{{\max\{c}_{g_{j}}(h-d),c_{g_{i}}(h-d)\}}\;+\;\frac{{x^{\left(3\right)}-\ell }_{a}-\ell_{d}}{c_{g_{j}}}\;-\;\Delta_{i},$$

(6b) $${t_{2}}_{\mathrm{ij}}^{\left(3\right)+}=\frac{\ell_{a}}{c_{g_{i}}}\;+\;\frac{\ell_{d}}{{\min\{c}_{g_{j}}(h-d),c_{g_{i}}(h-d)\}}\;+\;\frac{{x^{\left(3\right)}-\ell }_{a}-\ell_{d}}{c_{g_{j}}}\;+\;\frac{N}{f_{c}}\;+\;\Delta_{i},$$

where $c_{g_{j}}$ is the group velocity of the received mode, j, $c_{g_{i\mathrm{orj}}}\left(h-d\right)$ is the group velocity within the thinning region. It is necessary to consider velocity change at the thinning region because the SH1 mode is dispersive and its velocity is a function of the plate׳s thickness [4]. The minimum and maximum of the two possible modes within the thinner region length, in Eqs. (5a), (5b), (6a), (6b), is considered because, at first, both modes can propagate in the thinning region due to mode conversion of any incident mode at the leading edge, and the coefficients *TR_ij_* and *TT_ij_* consider the two possible modes, SH0 or SH1, propagating inside the thinning region. This, however, only holds when the region remaining thickness is above the SH1 mode cut-off thickness. Otherwise, its group velocity is not defined and this mode cannot propagate inside the thinning. Thus, either ${\min\{c}_{g_{j}}(h-d),c_{g_{i}}(h-d)\}$ or ${\max\{c}_{g_{j}}(h-d),c_{g_{i}}(h-d)\}$ should read $c_{g_{0}}\left(h-d\right)=c_{g_{0}}\left(h\right)=c_{g_{0}}$ in this case, since the group velocity for the non-dispersive SH0 mode is constant for any thickness. Also, in this case, a time gate for *T*_*i*1_ or *TR*_*i*1_ cannot be defined, therefore no time gate restriction was applied and the whole SH1 signal on the region is considered to calculate the *T*_*i*1_ coefficient, whereas *TR*_*i*1_ is not calculated in this case.

Prior to calculating the experimental reflection and transmission coefficients, it is necessary to compensate for attenuation. The experimental attenuation of the guided wave modes was calculated by receiving the SH0 and SH1 signals in several positions in a non-machined sample and fitting the peak-to-peak of the signals versus the position with an exponential decay curve. The exponential coefficient was then used to compensate the values of the amplitudes, $A_{i}^{\left(1\right)+},$ $A_{j}^{\left(1\right)-}$*,* $A_{j}^{\left(2\right)+}$*,* $A_{j}^{\left(2\right)-}$*and* $A_{j}^{\left(3\right)+}$ of the received signals considering the forward and backward propagating path at each position. Finally, the sixteen coefficients were calculated for each thinning depth and taper angle.

In addition to experiments, finite-element analysis was also performed using a commercial, time-domain, Finite-Element Method (FEM) solver, PZFlex©, which allows simulation of SH waves in two-dimensional models. The numerical model was executed for thinning depth from 0.5 to 7.5 mm in 0.5 mm step with the following taper angles, 90°, 65°, 55°, 45°, 35°, 30°, 25° and 10°, therefore including all the experimental thinning geometries. Numerical simulation mimicked the PPM EMATs generation by applying forces in surface nodes along the transducer length according to the transducer spatial profile following the procedure used and validated previously [3], [5], whereas reception was done by numerically convolving the wave field on the surface of the model with the probe spatial profile. Then, likewise in the experiments, the dual excitation and reception procedure, filtering and time gating were applied. Therefore numerical and experimental data can be straightforwardly compared. The only procedure which was not included in the numerical data was attenuation compensation since damping was not included in the model.

**Fig. 3:**
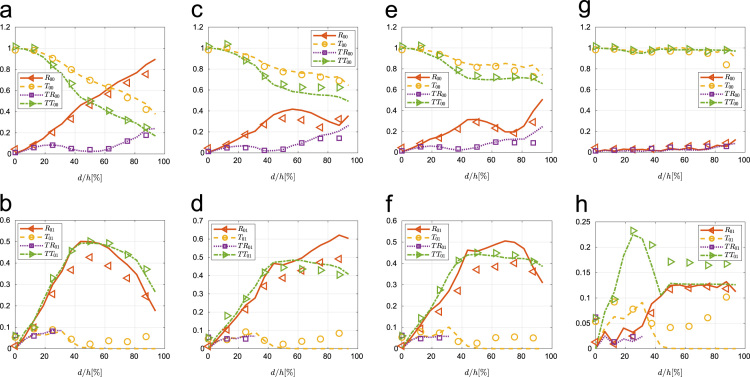
Coefficients for SH0 generation, obtained experimentally (symbols) and numerically (lines) at 90° edge (a) and (b), 55° (c) and (d), 45° (e) and (f) and 10° (g) and (h).

**Fig. 4:**
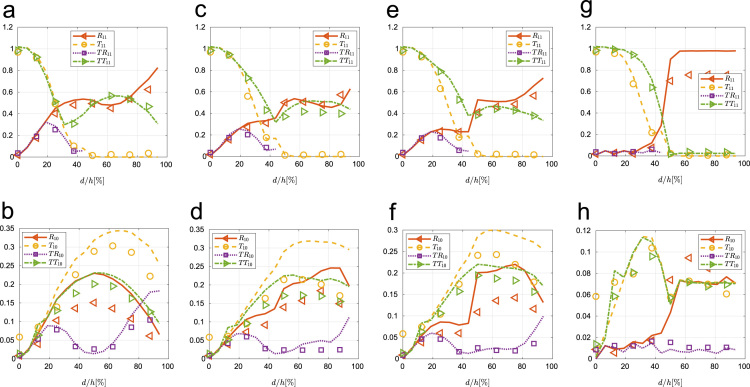
Coefficients for SH1 generation, obtained experimentally (symbols) and numerically (lines) at 90° edge (a) and (b), 55° (c) and (d), 45° (e) and (f) and 10° (g) and (h).

Figs. 3 and 4 show the experimental and numerical coefficients for generation of the SH0 and SH1 mode, respectively. This data not only helps on understanding the interaction of the SH0 and SH1 modes with wall thinning sections but also allows one to address the capabilities and limitation on the use of ultrasonic SH guided wave to estimate and detect wall thinning when both modes are allowed to propagate (see Ref. [1])*.*

## References

[1] Kubrusly A.C., Freitas M.A., von der Weid J.P., Dixon S. (2019). Interaction of SH guided waves with wall thinning. NDT E Int..

[2] Hirao M., Ogi H. (2003). EMATs for Science and Industry: Noncontacting Ultrasonic Measurements.

[3] Kubrusly A.C., Freitas M.A., Weid J.P. von der, Dixon S. (2018). Mode selectivity of SH guided waves by dual excitation and reception applied to mode conversion analysis. IEEE Trans. Ultrason. Ferroelectr. Freq. Control.

[4] Rose J.L. (2014). Ultrasonic Guided Waves in Solid Media.

[5] Petcher P.A., Dixon S. (2016). Mode mixing in shear horizontal ultrasonic guided waves. Nondestruct. Test. Eval..

