# Supplementary material for: Dataset on reflection and transmission coefficients of ultrasonic shear horizontal guided waves in plates with wall thinning
Source: Data Brief. 2018 Nov 15;21:2179–91. doi: 10.1016/j.dib.2018.11.053 (PMC6265498; doi:10.1016/j.dib.2018.11.053)
Supplement: Supplementary file 1 — Supplementary material [file mmc1.docx]

Dear editor,

We wish to confirm that there are no conflicts of interest associated with this publication (DIB-D-18-02834) entitled “Dataset on reflection and transmission coefficients of ultrasonic shear horizontal guided waves in plates with wall thinning”.

Yours faithfully,

Alan Conci Kubrusly

(On behalf of the authors of the manuscript)
